# UV Light Inhibited HRV1b Replication but Reduced Adherens Epithelial Junction and Antiviral Responses via SOCS1 in Human Respiratory Epithelial Cells

**DOI:** 10.3390/v18030303

**Published:** 2026-02-28

**Authors:** Jeba Maimuna, Zuqin Yang, Elke Bachmann, Susanne Mittler, Sonja Trump, Susetta Finotto

**Affiliations:** Department of Molecular Pneumology, Friedrich-Alexander-Universität (FAU) Erlangen-Nürnberg, Universitätsklinikum Erlangen, Hartmannstraße 14, 91052 Erlangen, Germany

**Keywords:** nasal epithelial cells (NECs), rhinovirus, UVC light, IR light, epithelial cell barrier, asthma, SOCS1, interferon

## Abstract

Human rhinovirus (HRV) is one of the common respiratory viral infection agents that triggers airway obstruction and asthma exacerbations, especially during childhood. This project aimed at evaluating the mechanism of ultraviolet (UV) and infrared (IR) radiations to inactivate HRV infection and replication inside and outside infected airway epithelial cells and the resulting impact on interferon responses and epithelial barrier integrity. Hereby, airway epithelial cells were infected with different RV concentrations. Later these cells are exposed to UV and IR light to analyze their impact on the viral immune response of the host by real-time PCR. It was found that RV1B disrupted cell junctions of airway epithelial cell barriers. Moreover, high doses of RV1B activated pattern recognition receptor (TLR3), induced interferon (IFN-β) response and reduced SOCS1, which is a negative regulator of IFN-β. Further, IR lights inhibited rhinovirus post infection in primary nasal epithelial cells (NECs). Finally, UVC exposure significantly inhibited the antiviral effects of the host via SOCS1 inhibition and decreased RV1B within 72 h. Collectively, these findings support the role of UV light as an effective therapeutic approach for acutely eliminating RV but resulting in barrier and antiviral damage, which can have a drawback effect for asthma.

## 1. Introduction

Asthma is a chronic inflammatory disease of the airways. This inflammation causes bronchial hyperreactivity, bronchospasm and mucus overproduction, leading to lung obstruction. These complications lead to recurrent episodes of breathlessness and wheezing due to air trapping and increased dead space [1].

Asthma is typically considered to be either an allergic or non-allergic disease. Allergic asthma, also known as TH2 asthma, often occurs in childhood and is associated with induced pathological TH2 cell responses in the airways. Allergic asthma can be triggered by exposure to environmental allergens such as house dust mites, pollen and animal dander, or by new allergens (e.g., occupational allergens) in later life [2].

Non-allergic or late onset asthma is more severe and less likely to be allergy related than childhood asthma. The tendency for excessive IgE production and CD4 + TH2 differentiation due to an immune response to various antigens or allergens is called atopy [3]. In children, atopy, lower lung function and respiratory infections, especially those caused by rhinoviruses, are the most important risk factors for persistent asthma [2].

The airway epithelium is a continuous, pseudostratified group of cells that line the respiratory tract and provide a barrier function to inhaled gasses, particulate matter, pathogens and other xenobiotics [4]. In the healthy state, the airway epithelium is a highly regulated structure composed of tightly bound epithelial cells. It acts as a physical and immune barrier to external influences. However, environmental stimuli can potentially damage the epithelium and expose the underlying submucosal layer, leading to chronic pathological changes such as airway remodeling.

The respiratory tract contains a number of different types of epithelial cells, including cilia, goblet, and basal cells [5]. Each of these cells has a distinct set of properties that give the bronchial epithelium its sophisticated functionalities. Ciliated cells transport tracheobronchial secretions in the airway tract and form the primary host defense barrier. The goblet cells synthesize mucin and produce a layer of mucus that helps to trap pathogens and debris in the respiratory tract. Basal cells adhere to surface cells and differentiate into ciliated cells that purify the airways as part of regular epithelial turnover and repair in the distal airways [5].

These cells play a vital role in removing and neutralizing toxic and irritant substances from the inhaled air by preventing them from reaching the alveoli, where vital gas exchange takes place [6]. The defense mechanism of airway epithelial cells is achieved through the secretion of antimicrobial peptides by airway epithelial cells (AECs) and the regulation of barrier integrity by intracellular apical junctional complexes (AJCs). AJCs are composed of TJs and AJs that connect neighboring cells through interactions between membrane-spanning adhesion molecules. Tight junctions control epithelial barrier function and are associated with the expression of intracellular adhesion complexes. These complexes regulate the passage of substances (macromolecules, water and ions) between cells. At the cellular level, TJs are associated with cytoplasmic scaffolding proteins like zonula occludens (ZO-1, ZO-2, and ZO-3) and cingulin, and are attached to the actin cytoskeleton [7]. AJs are composed of two major families of transmembrane proteins: the cadherins (such as E-cadherin) and the nectins. At the intracellular level, AJs attach to a cytoplasmic scaffold and signaling complex consisting of the p120 catenin, β-catenin and α-catenin proteins. The absence of E-cadherin from lung epithelia leads to loss of ciliated cells, epithelial exposure and suppression of club cell differentiation, which hinders epithelial healing after injury due to the stem cell properties of club cells [8]. Respiratory viruses can interfere with TJs and AJs by regulating protein expression or localization of their components [9]. RV is one of the common respiratory viral infections that trigger airway obstruction and exacerbations in both children and adults when asthma is already established [10]. The epithelial cells of the respiratory tract are the primary site of infection with HRV [11].

Human rhinovirus (HRV), a non-enveloped, positive-sense, single-stranded RNA respiratory virus of the family Picornaviridae, is the leading cause of upper respiratory tract infections worldwide. HRV has 11 viral proteins, four of which (VP1, VP2, VP3 and VP4) form the viral capsid, and the rest are responsible for viral replication and assembly. There are three types of HRV (HRV-A, HRV-B and HRV-C). Types A and C are the most commonly reported species in children with respiratory infections, whereas HRV-B is associated with mild respiratory illness or asymptomatic presentation [12].

HRV-A, HRV-B and HRV-C bind to specific receptors on host cells. The majority of RVs in the major group of RV-A and RV-B species bind to intercellular adhesion molecule-1 (ICAM-1), while a subset of RVs in the minor group of RV-A species target the low-density lipoprotein receptor (LDLR). The minor group of HRV-A species includes 1A, 1B, 2, 29, 30, 31, 44, 47, 49, 62, which target LDL receptors for cellular entry, whereas RV-C uses the cadherin-related family member 3 (CDHR3) protein as its receptor [13,14]. As this group represents the majority of RVs involved in human disease, we have used the minor group RV1B in our studies.

Rhinovirus can disrupt the function of tight junctions and the epithelial barrier of the lung, particularly on the alveolar side of the alveolar–capillary barrier [15]. Alterations in TJs are often characterized by reduced TJ strand formation, strand breaks, and altered TJ protein expression and distribution [15], which can lead to increased epithelial permeability, facilitating viral invasion and triggering inflammatory responses [16]. HRV also affects AJ proteins, leading to a reduction of transepithelial resistance and increased permeability of the epithelial barrier to macromolecules [17].

HRV enters host cells by a process called endocytosis, facilitated by LDLR receptors. Once inside the host cells, HRV is recognized by pattern recognition receptors (PRRs) in the airways [18]. Pattern recognition molecules such as toll-like receptors (TLRs) are expressed by epithelial cells. TLR3 is a member of the TLR family of proteins that is localized to the endosomal compartment of myeloid DCs, whereas it is both expressed intracellularly and on the cell surface of fibroblasts and epithelial cells. TLR3 is thought to be a receptor for dsRNA. When TLR3 recognizes dsRNA, it transmits signals via the adaptor protein Toll-IL-1 receptor (TIR) domain-containing adaptor molecule-1 (TICAM-1) (also known as TIR domain-containing adaptor inducer IFN-β [TRIF]) [19]. This leads to activation of NF-κB and induction of type I IFNs (particularly IFN-β) [20]. TLR3 is effective in the identification of pathogens and the triggering of pro-inflammatory responses, including the release of chemokines for the recruitment of phagocytes and other cells [21]. TLR3 ligand is present in lung epithelial cells and is stimulated by polyinosinic:polycytidylic acid (poly(I:C)). It has been shown that virus-induced severe asthma is exacerbated by the detection of dsRNA. Viral infection induces IFN-α/β and IFN-inducible genes, which are important in antiviral host defense, and epithelial cells also initiate host defense responses such as the production of antiviral molecules.

The viral pathogen causes inflammation, which results in the upregulation of TLR3 expression. The pattern recognition receptor TLR3 enables healthy airway epithelial cells (EpCs) to recognize and respond to rhinovirus [22]. TLR3 induces an antiviral response (IFN-β) against rhinovirus by enhancing the innate immune arm of the host response. Viral infection triggers the expression of IFN-α/β and IFN-inducible genes, which play a crucial role in antiviral host defense. Insufficient TLR3 expression may be associated with defective IFN-β responses and increased susceptibility to HRV infection in asthmatics [23].

After a viral infection, IFN-β are produced, which activate the JAK-STAT pathway. The JAK-STAT pathway activates the expression of toll-like receptor (TLR)-3, interferon regulatory factor (IRF)-7 and monocyte inflammatory protein (MIP)-1 alpha [24]. However, this signaling pathway is inhibited by SOCS1 (a potent member of the SOCS family), which is a key regulator of a number of cytokines involved in the immune response [24]. SOCS1 acts as a negative feedback inhibitor of cytokine-induced signaling through the JAK/STAT pathway and plays an important role in the antiviral response [25].

The sun is the main source of ultraviolet radiation (UVR), a type of non-ionizing radiation in the electromagnetic spectrum. It emits UVA (320–400 nm), UVB (280–320 nm) and UVC (<280 nm) radiation [26]. UVA accounts for the majority of ultraviolet radiation reaching the earth’s surface, with only 5% being absorbed by the ozone layer. In contrast, 95% of UVB and 100% of UVC are absorbed in the upper stratosphere [27]. High-energy UV light can be absorbed by the bases in RNA and DNA, resulting in the fusion of two adjacent pyrimidines into covalently linked dimers that are unable to properly pair. Low-energy UV light can also lead to the formation of pyrimidine dimers and may cause further genetic damage by forming reactive oxygen species that oxidize bases and break strands [28]. UVA radiation is well absorbed in the dermis and the underlying subcutaneous fat, while UVB radiation penetrates the epidermal layer and the upper surface of the dermis [29]. Compared to UVB and UVA, UVC, which is absorbed by nucleic and amino acids, has the strongest antimicrobial properties and the most damaging effect on nucleic acids [30]. UVC disinfection does not kill pathogens. Instead, it inactivates them, preventing reproduction and infection [31]. Exposure to UVC light typically causes dimerization between two consecutive thymine residues, thereby destroying the hydrogen bonds between thymine and adenine and forming a new thymine–thymine dimer [27]. However, depending on the specific nucleic acid, other dimers may also be formed, including cytosine–cytosine, cytosine–thymine, uracil–uracil, uracil–thymine and uracil–cytosine dimers. It crosslinks thymine and cytosine (pyrimidine nucleoside bases), two pyrimidine nucleoside bases in the same DNA strand, resulting in unpaired bases. This leads to the formation of cyclobutylpyrimidine dimers (CPDs), which disrupt DNA replication, transcription and translation, affecting cellular function and ultimately inactivating viruses [32]. UVA, UVB, and UVC all generate these dimers, which disrupt crucial biological processes and cause immunosuppression or viral inactivation. The ability of UV light to neutralize microbes is dependent on the intensity, wavelength and duration of exposure, with shorter wavelengths being more effective. It is demonstrated that UV light has long been shown to be effective against several strains of airborne viruses and is a direct antimicrobial approach [33].

Infrared (IR) heating involves the transmission of thermal energy in electromagnetic waves [34]. IR can be used to generate high temperatures, as high temperatures are considered a classic method of sterilization [35]. IR is categorized into near infrared (NIR) (IR-A; 700–1400 nm), medium infrared (IR-B; 1400–3000 nm) and far infrared (IR-C; 3000 nm–1 mm) [36]. The physical principles of NIR are the same as those of visible light. Infrared radiation type A (IR-A) or NIR has the ability to penetrate both the epidermal and dermal layers of the skin and reach the subcutaneous tissue with minimal increase in skin temperature. A wide range of viruses have been inactivated by NIR or IR-A light [37]. In contrast, type B (IR-B) and C (IR-C) infrared radiation are mainly absorbed by the epidermal layers of the skin. IR exposure induced DNA damage, but surprisingly did not disrupt normal epithelial cell differentiation or integrity [38]. Compared to traditional heating methods, infrared (IR) heating has many advantages. These include faster heating times, uniform heat distribution, reduced quality loss, flexibility, ease of use, light and compact equipment, and significant energy savings [39].

The principal aim of this work is to examine the mechanism of UV and IR to inactivate RV1B, RV1B-infected cells and the resulting impact on interferon response and epithelial barrier integrity.

In this project, the A549 cell line was used because the primary site of RV1B infection and replication in both the upper and lower airways is the human airway epithelial cells. Moreover, the A549 cell line is a well-established in vitro model for studying airway viral infection due to its high acceptance to broad spectrum respiratory viruses and its use for innate antiviral signaling [40]. Besides the A549 cell line, NECs were also used as primary cells. Hereby, different RV concentrations and different time points were used to infect the A549 cell line and NECs, later treated by UV and IR to analyze the amount of intracellular and extracellular RV mRNA by qPCR.

RV is an important human pathogenic agent causing a range of diseases, including asthma exacerbations, especially in children [41]. There is a high chance of developing persistent childhood asthma when children develop RV-induced wheezing and respiratory allergies at an early age. To limit airborne viral transmission, inactivating the virus production for a short time can be an option. It has been reported that germicidal light technologies like UV and IR radiations have antimicrobial properties. Therefore, this study leads to a better understanding of the RV1B effect on the epithelial barrier integrity as well as its antiviral function (interferon responses) through evaluating underlying mechanisms of germicidal light treatments like UV and IR.

Despite the rising prevalence of asthma, current therapies and medications only address the symptoms and have high side effects. Furthermore, this study aims to establish better therapeutic interventions with fewer side effects for asthma patients.

## 2. Materials and Methods

All of the experiments described in this manuscript were performed in compliance with locally approved ethical guidelines. These experiments were performed at the Department of Molecular Pneumology, University Hospital Erlangen, Germany.

### 2.1. Human Cohort Study “AZCRA”

The human cohort study “AZCRA” (Investigation of the role of cytokines, chemokines, and their receptors in the inflammatory process in asthma patients) was conducted at the University Hospital Erlangen and was approved by the ethics review board of the Friedrich-Alexander University of Erlangen–Nuremberg (315_20B). The study was registered in the German Clinical Trial Register (DRKS00023843). Asthmatic patients atopic and non-atopic, and non-asthmatic control patients between 18 and 65 years of age, were recruited, and informed written consent was obtained. At the Baseline Visit B0, during a non-symptomatic phase, nasal swabs were taken. General clinical characteristics describing the control and asthma subjects are reported in Table 1. Briefly, in this study we analyzed the NECs of 3 subjects. Subject number 1 is 65 years old with atopic dermatitis; subject number 2 is 27 years old and has asthma; and the third one is 32 years old and does not have asthma.

### 2.2. Primers

The following primers were used (Table 2): HPRT (hypoxanthine-guanine phosphoribosyl transferase), RPL30 (ribosomal protein L30), RV1B (rhinovirus 1B), ZO-1 (zonulin-1), E-cadherin, TLR3 (toll-like receptor 3), SOCS1 (suppressor of cytokine signaling 1), which were purchased from Eurofins Scientific Group, Luxemburg.

### 2.3. Cell Culture

After the thawing process, A549 lung adenocarcinoma cells were expanded in RPMI 1640 medium (Anprotec, Bruckberg, Bavaria, Germany) supplemented with 10% FCS, 5% PenStrep, and 5% L-Glutamine and maintained under standard condition at 37 °C and 5% CO_2_ until they gained 70–80% confluency. After reaching the desired confluency, both A549 cells and NECs were washed with PBS (Anprotec, Bruckberg, Bavaria, Germany) and detached with trypsin-EDTA (Anprotec, Bruckberg, Bavaria Germany) and plated in 2 mL medium in a 6-well plate.

In the case of NECs, nasal cells were sampled with a nasal swab from the nasopharyngeal fluid of the three patients at the molecular pneumology lab, University Hospital Erlangen (Table 1). These cells were collected from the swabs by centrifugation (450× *g*, 5 min, 4 °C) and treated with DNAse I (1.5 mg/mL, diluted in H_2_O; Sigma-Aldrich, St. Louis, MO, USA) for 20 min and washed with medium afterwards. Then, depending on the condition, the nasal epithelial cells (NECs) were freshly infected with RV-1B (protocol below) or directly seeded into collagen-coated 6-well plates in 2 mL of 50% Pneumocult+ medium (PneumoCult-Ex-Plus Medium (Stemcell™, Grenoble, France) + 1% Antibiotic–Antimycotic (Gibco™, Waltham, MA, USA) and 0.5% Gentamicin (10 mg/mL; Sigma-Aldrich, St. Louis, MO, USA), and 50% Pneumocult+ medium (Pneumocult+ medium +5% FBS (Sigma-Aldrich, St. Louis, MO, USA) +2% Sodium Bicarbonate (Gibco^TM^, Waltham, MA, USA)). At the end of incubation the cells were harvested, and RNA was extracted for qPCR analysis and RV detection. A typical representative nasal epithelial cell characterization is shown in Supplementary Figure S1. 

### 2.4. RV1B Infection

The RV1B suspension was thawed at room temperature at first. Specific RV1B suspensions were added to each condition of cell pellets (0.125 mL RV1B, 0.25 mL RV1B, 0.5 mL RV1B, 0.25 mL RV1B + UV, 0.25 mL RV1B + IR) (conditions varied depending on experimental protocol/design). Then, the infected cells were incubated at 33 °C for one hour at 500 rpm on a shaker TH15 (Edmund Buehler GmbH, Bodelhausen, Germany). Afterwards, specific media with their necessary supplements were added to cells to stop the reaction. After discarding the supernatant, the different conditioned A549 cells transferred to the 6-well plates directly for 48 h and 72 h. For the “uninfected” condition, 500 μL of A549 cells were added to a selected amount of RPMI 1640 medium in each “uninfected” condition of the 6-well plates and incubated for 48 h and 72 h.

NECs were centrifuged at RT for 15 min at 300 *g*, and the supernatant was aspirated. Next, specific RV1B suspensions were added to each condition of cell pellets and incubated at 33 °C for one hour at 500 rpm on a shaker. After that, each condition was mixed with 5 mL of media and centrifuged at RT for 15 min at 300× *g*. The supernatants were discarded. A total of 3 mL Pneumacult + and 3 mL pneumacult + were added to each condition and resuspended well. A total of 2 mL of NECs were then transferred to previously prepared collagen-coated 6-well plates. Finally, they were incubated for 72 h. NECs were transferred to previously collagen-coated 6-well plates. NECs were incubated for 72 h.

### 2.5. UV Irradiation

Six-well culture plates containing A549 cells and NECs were placed under a hood and exposed to UV irradiation for 30 min to prepare all “post-infected RV1B” conditions. According to the experimental protocol, they were then removed from the hood and incubated separately for 48 and 72 h.

For the preparation of the “UV pre-infected 0.25 mL RV1B” condition, the RV1B was first thawed at RT and aliquoted into a 2 mL eppendorf tube. The tube was then exposed to UV light for 30 min. After this treatment, the UV-treated RV1B was used to infect the 0.25 mL RV+UV conditioned A549 cells and NECs.

### 2.6. Infrared Irradiation

A single illumination source with a wavelength range of 780–1400 nm, covering the entire near-infrared (NIR)/IR-A region, was used to expose A549 cells and NECs to infrared light IR11 (Petra Electric, Burgau, Bavaria, Germany). To ensure uniform illumination across the culture plates, the infrared light source was placed 22 cm above the cell-containing 6-well culture plates. Exposure was programmed for 10 min every 12 h for an overall duration of 48 h.

In the case of the “IR pre-infected 0.25 mL RV1B + IR” condition, the RV1B was first thawed and then aliquoted to 2 mL in a 2 mL Eppendorf tube. The aliquoted RV was treated with infrared light for 10 min. The IR-treated RV was then used to infect the “0.25 mL RV1B + IR” conditioned A549 cells and NECs.

### 2.7. RNA Isolation and Reverse Transcription Polymerase Chain Reaction (RT-PCR)

RNA was isolated from cells by following three steps: phase incubation, RNA precipitation and RNA washing. RNA concentration was measured using Nanodrop software (NanoDrop 2000/2000C Operating Software from Thermo Fisher Scientific, Waltham, MA, USA) in Nanodrop 2000C (Peqlab, Erlangen, Germany). RNA was used for cDNA synthesis. In order to synthesize cDNA for each sample, a master mix consisting of 4 µL 5x reaction buffer, 2 µL dNTP mix, 1 µL RiboLock RNase inhibitor, 1 µL reverse transcriptase, and 1 µL random hexamer primer was prepared. In addition, 1 µg of RNA was added, and each probe was filled to 11 µL with sterile water. The RNA was then translated into cDNA using the master cycler (PeQlab, Erlangen, Germany) according to the protocol (5 min at 25 °C, 60 min at 42 °C, 5 min at 70 °C).

The qPCR was carried out using a master mix for each probe. The master mix constitutes 10 μL SYBR green, 5 μL primer, and 2 μL DEPC H20. A total of 17 µL of master mix and 3 µL of cDNA for each probe were loaded onto qPCR plates (Bio-Rad Laboratories, Inc. Hercules, CA, USA). The qPCR plates were loaded into a C1000TM thermal cycler (BIO-RAD Laboratories, Hercules, CA, USA). The DNA was amplified using the BioRad CFX Maestro software version number 2.3, and the protocol was followed (105 °C, 30 min at 94 °C, 30 min at 50 °C, 2 min at 72 °C). The results of all the primers and the viral load (RV) were then analyzed by plotting the housekeeping genes (HPRT and RPL30) against each of the primers using GraphPad.

### 2.8. Immunofluorescence and Confocal Microscopy

Microscope slides (76 × 26 mm) were marked with a liquid blocker to block fluids. To fix the A549 cells in microscopic slides and cytospins, 100 μL of 4% PFA was initially added to the marked position RT and left for 15 min. The cells were then washed three times with PBS. After washing, a permeabilization buffer (0.25% TritonX in PBS; Invitrogen, Carlsbad, CA, USA) was applied and left for 10 min. The cells were subsequently washed three times with PBS. Blocking buffer (5% Bovine albumin/BSA in PBS) was then added and left at RT for 30 min. RV VP3 (1:50; Invitogen, Carlsbad, CA, USA) in 1% BSA/PBS was then added at RT and kept for 1 to 2 h. The A549 cells were then washed three times with PBS. AlexaFluor 555 (1:500; Invitrogen, Carlsbad, CA, USA) in 1% BSA/PBS was then added to the cells at RT for 1 h, and the cells were again washed three times with PBS. DAPI (1:5000) was then added to the cells at RT for 10 min, followed by three washes with VE H20. Finally, the A549 cells were covered with FluorSave mounting medium (Merck, Darmstadt, Hesse, Germany) and stored until microscopic analysis.

Cells were analyzed following the manufacturer’s instructions using an inverted Zeiss microscope Observer D1 (Oberkochen, Baden-Württemberg, Germany) by using the Zen Blue software version 2.0 (Zeiss, Oberkochen, Baden-Württemberg, Germany). All images were taken at a magnification of 63x and are presented as the maximum projection of the image.

### 2.9. Statistical Analysis

The statistical analysis was carried out using GraphPad Prism, version 9.0. The figure legend for each figure details the number of subjects and statistical analyses used. All data are presented as standard error of the mean (SEM). All correlations were statistically compared by one-way ANOVA and analysis of variance (ANOVA), with Tukey’s post hoc test used for multiple comparisons for one-way ANOVA. Statistically significant values were taken as * = *p* < 0.05, ** = *p* < 0.01, *** = *p* < 0.001, **** = *p* < 0.0001 and were assigned in specific figures and experiments as shown. All differences were considered significant if *p* ≤ 0.05.

## 3. Results

### 3.1. IR Treatment Did Not Inhibit RV1B Replication in A549 Cells

**Figure 1:**
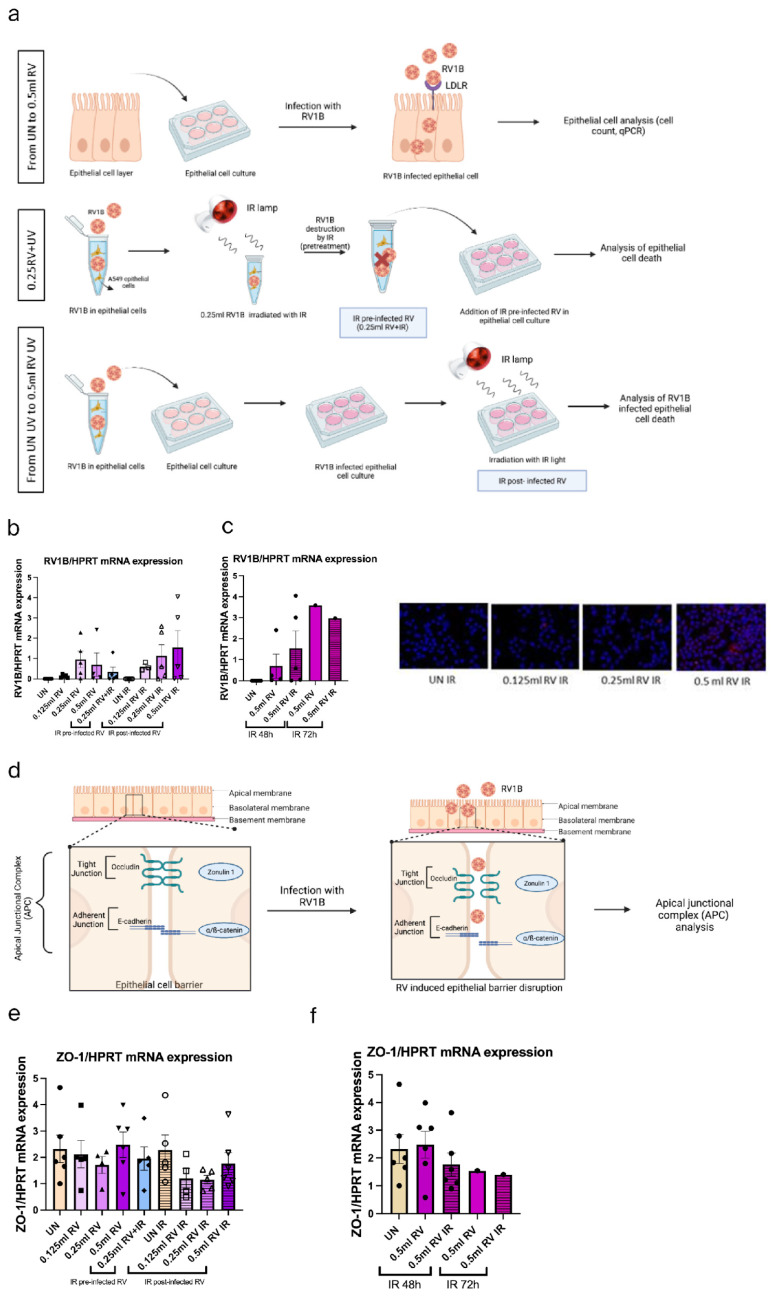
IR treatment did not inhibit RV1B replication in airway epithelial tumor cell line A549. (**a**) Schematic illustration of RV1B infection in epithelial cells. IR pre-irradiated and post-irradiated RV-infected airway epithelial cells. Effects of RV infection in untreated RV mRNA and IR-irradiated RV RNA in A549 epithelial cells were measured within 48 h with immunostaining results. Merged images shown (VP3 = pink; DAPI = blue). Data represents six independent observations per group, representative of two independent experiments. (**b**,**c**) Effects of untreated and IR-irradiated A549 epithelial cells on RV mRNA measured within 72 h. (**d**) Schematic illustration of epithelial barrier integrity via observing tight junctions in RV-infected, IR light pre-irradiated and post-irradiated RV-infected airway epithelial cells. (**e**) Effects of tight junction in untreated and IR-irradiated RV-infected A549 epithelial cells were measured within 48 h. Each circular dot represents one independent sample per experiment and the number of dots per group corresponds to the sample size. Data represents six independent observations per group, representative of two independent experiments. (**f**) Effects on tight junction in untreated and IR-irradiated RV-infected A549 epithelial cells were measured within 48 h and 72 h. The mRNA levels of each condition were determined using real-time polymerase chain reaction. (**a**,**d**) Created in BioRender.com.

IR light, especially near-infrared light, has significant anti-inflammatory therapeutic effects [42]. A549 cells were either infected with increasing concentrations of RV1b and left untreated for 48 or 72 h or RV-infected and then treated with mild IR treatment for 10 min, as shown in Figure 1a. Furthermore, we infected A549 with inactivated RV-IR (IR pre-infected RV), which can be found as a positive control.

RV1b mRNA expression was induced after both RV and IR 48 h treatment, which confirmed the disability of IR treatment in RV destruction (Figure 1b). Moreover, no significant reduction of RV1b mRNA expression was observed after IR 72 h treatment (Figure 1c). Taken together these data suggested that IR treatment could not destroy RV in epithelial cells.

Next, to explore the effect of RV and IR on the tight junction (Zonulin-1), we investigated ZO-1 mRNA expression levels under RV and IR treatment (Figure 1d–f). Here, we observed minimal reduction of Zo-1 mRNA expression after IR treatment. This data suggests that IR inhibits tight junctions in airway epithelial cells slightly at 48 h.

### 3.2. IR Treatments Show Reduction of Antiviral Function at 48 h in A549 Cells

**Figure 2:**
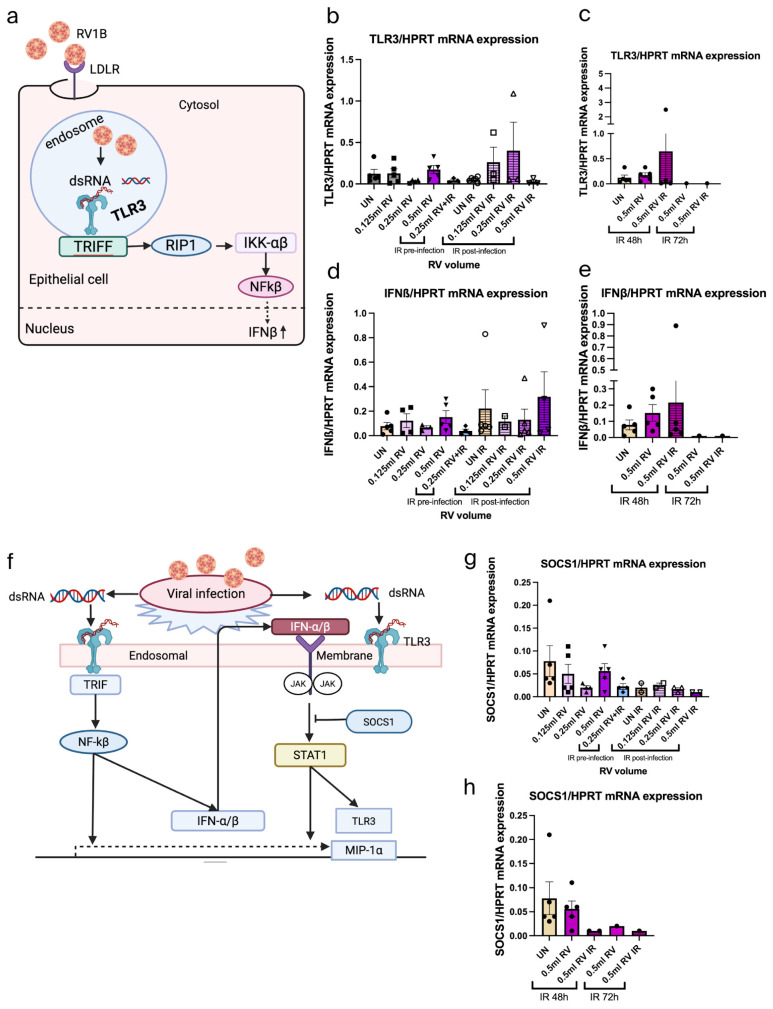
IR treatment shows low induction of antiviral response in lung carcinoma epithelial cell line A549 at 48 h. (**a**) Schematic illustration of RV1B infection in the viral recognition capability and antiviral response of airway epithelial cells. (**b**) Effects of viral sensing capability in untreated and IR-irradiated RV-infected A549 epithelial cells measured within 48 h. Data represents six independent repeats per group, representative of two independent experiments. (**c**) Effects of viral recognition in untreated and IR-irradiated RV-infected A549 epithelial cells measured within 72 h. (**d**) Effects of antiviral response in untreated and IR-irradiated RV-infected A549 epithelial cells measured within 48 h. Data represents six independent repeats per group, representative of two independent experiments. (**e**) Effects of antiviral response in untreated and IR-irradiated RV-infected A549 epithelial cells measured within 72 h. (**f**) Schematic illustration of the inhibitory effect of SOCS1 in JAK/STAT pathway in airway epithelial cells after RV1B infection. (**g**) SOCS1 expression in untreated and IR-irradiated RV-infected A549 epithelial cells measured within 48 h. Data represents six independent repeats per group, representative of three independent experiments. (**h**) SOCS1 expression in untreated and IR-irradiated RV-infected A549 epithelial cells measured within 72 h. The mRNA levels of each condition were determined using real-time polymerase chain reaction. Each circular dot represents one independent sample per experiment and the number of dots per group corresponds to the sample size. (**a**,**f**) Created in BioRender.com.

To address the antiviral function in A549 cells under IR treatment, we have further investigated the TLR3 expression for sensing the viral infection, IFN-β response for understanding the antiviral response, and SOCS1 expression to understand the negative regulation of IFN-β response. Analysis or TLR3 expression demonstrated low induction of TLR3 expression after IR 48 h treatment (Figure 2a–c). Moreover, the interferon response also shows the same result as the TLR3 expression where the IFN-β expression was induced in RV-infected epithelial cells after IR 48 h treatment (Figure 2d,e). Lastly, the SOCS1 expression was analyzed after IR 48 h and IR 72 h treatment where SOCS1 mRNA expression was reduced (Figure 2f–h). Combined, this data supports the notion that IR treatment does not induce the antiviral response in RV-infected epithelial cells at 48 h and 72 h.

### 3.3. IR Inhibited RV1B Without Affecting Tight and Adherens Junctions in Primary Nasal Epithelial Cells (NECs)

**Figure 3:**
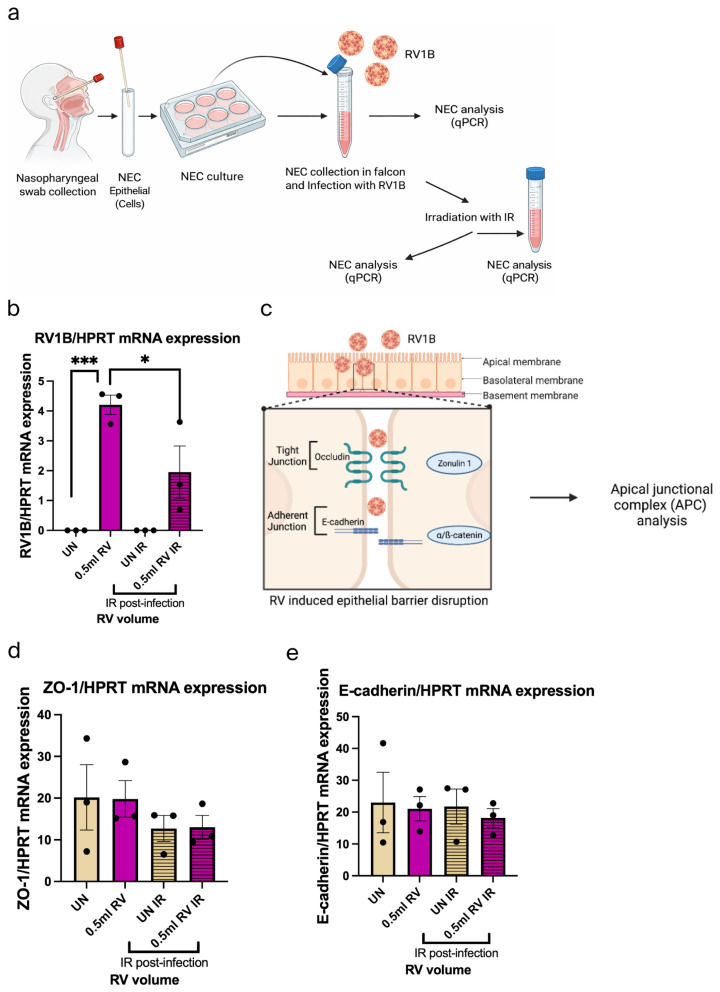
IR inhibits RV1B and maintains tight and adherens junctions in primary nasal epithelial cells (NECs). (**a**) Experimental design of RV1B infection in NEC culture and IR irradiation. (**b**) Effects of RV infection in untreated and IR irradiation on RV RNA in NECs within 72 h. (**c**) Schematic illustration of RV1B infection in the barrier junctional proteins of airway epithelial cells. (**d**) Tight junctions mRNA expression in untreated and IR-irradiated RV-infected NECs measured within 72 h. Data represents three patients per group, representative of one independent experiment. (**e**) Adherens junctions expression in untreated and IR-irradiated RV-infected NECs measured within 72 h. Data represents three patients per group, representative of one independent experiment. The mRNA levels of each condition were determined using real-time polymerase chain reaction. IR reduced the RV infection in NECs but could not inhibit it totally. One-way ANOVA analysis with Tukey’s post hoc test was performed to test statistical significance. *p* values * *p* < 0.05; *** *p* < 0.0001. Each circular dot represents one independent sample per experiment and the number of dots per group corresponds to the sample size. (**a**,**c**) Created in BioRender.com.

The role of IR light on RV infection in NECs derived from three subjects was analyzed. Here, their NECs were either infected with 0.5 mL RV1B and left untreated for 72 h or 0.5 mL RV-infected and then treated with IR light for 10 min, as indicated in Figure 3a. This treatment revealed that RV was induced significantly after the RV infection in NECs, whereas it was significantly reduced after IR 72 h treatment (Figure 3b).

Furthermore, we observed the effect of RV and IR on the tight junction (Zonulin-1) and adherens junctions (E-cadherin) in NECs (Figure 3c–e). We did not observe any significant change after RV infection and IR treatment, which indicated that IR did not influence tight and adherens junctions in NECs.

### 3.4. IR 72 h Treatment Has Small Viral Sensing Capability and Antiviral Response in RV-Infected Nasal Epithelial Cells

**Figure 4:**
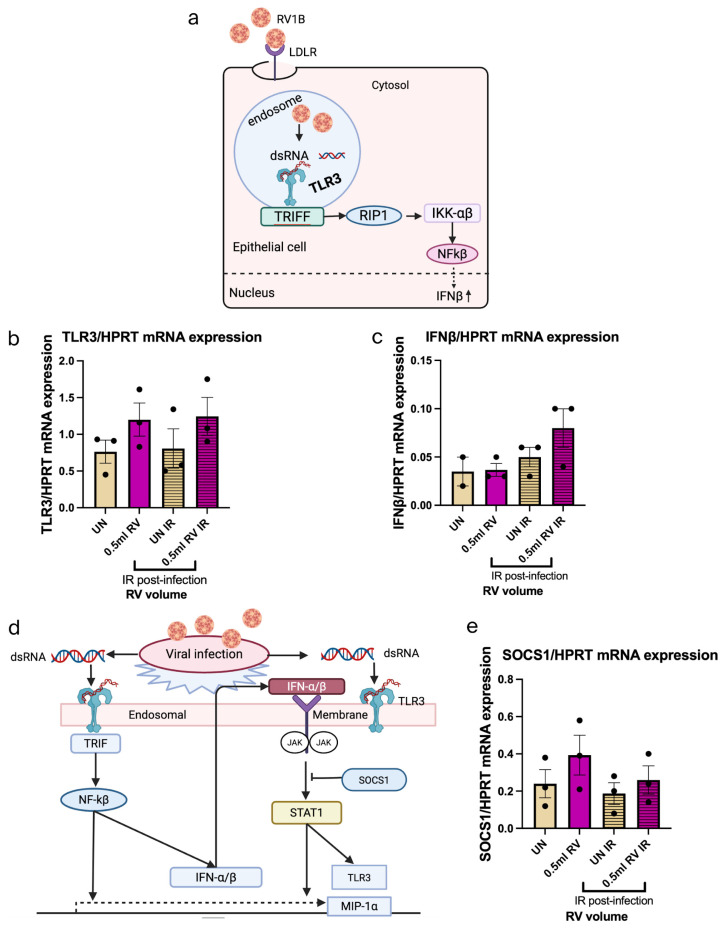
IR 72 h treatment induces antiviral response in RV-infected nasal epithelial cells. (**a**) Schematic illustration of RV1B infection in the viral recognition capability and antiviral response of airway epithelial cells. (**b**) Effects of viral sensing capability in untreated and IR-irradiated RV-infected NECs measured within 72 h. Data represents three patients per group, representative of one independent experiment. (**c**) Effects of antiviral response in untreated and IR-irradiated RV-infected NECs measured within 72 h. Data represents three patients per group, representative of one independent experiment. (**d**) Schematic illustration of the inhibitory effect of SOCS1 in JAK/STAT pathway in airway epithelial cells after RV1B infection. (**e**) SOCS1 expression in untreated RV mRNA and IR-irradiated RV mRNA in NECs measured within 72 h. Data represents three patients per group, representative of one independent experiment. The mRNA levels of each condition were determined using real-time polymerase chain reaction. Each circular dot represents one independent sample per experiment and the number of dots per group corresponds to the sample size. (**a**,**d**) Created by BioRender.com.

After RV infection and IR 72 h treatment, we analyzed the TLR3 expression and IFN response in NECs (Figure 4a–c). Interestingly, this data indicated small induced TLR3 expression after both RV infection and IR treatment. Besides that, IFN-β mRNA expression was induced by trend after IR-treated RV-infected NECs. SOCS1 mRNA expression was analyzed after RV infection and IR treatment in NECs (Figure 4d,e). We found that SOCS1 was induced by trend after RV infection but reduced after IR treatment, explaining the rise in IFN-beta.

### 3.5. Antiviral Response Induced by RV1B Was Inhibited by UV Through Elimination of RV1B in Nasal Epithelial Cells (NECs)

**Figure 5:**
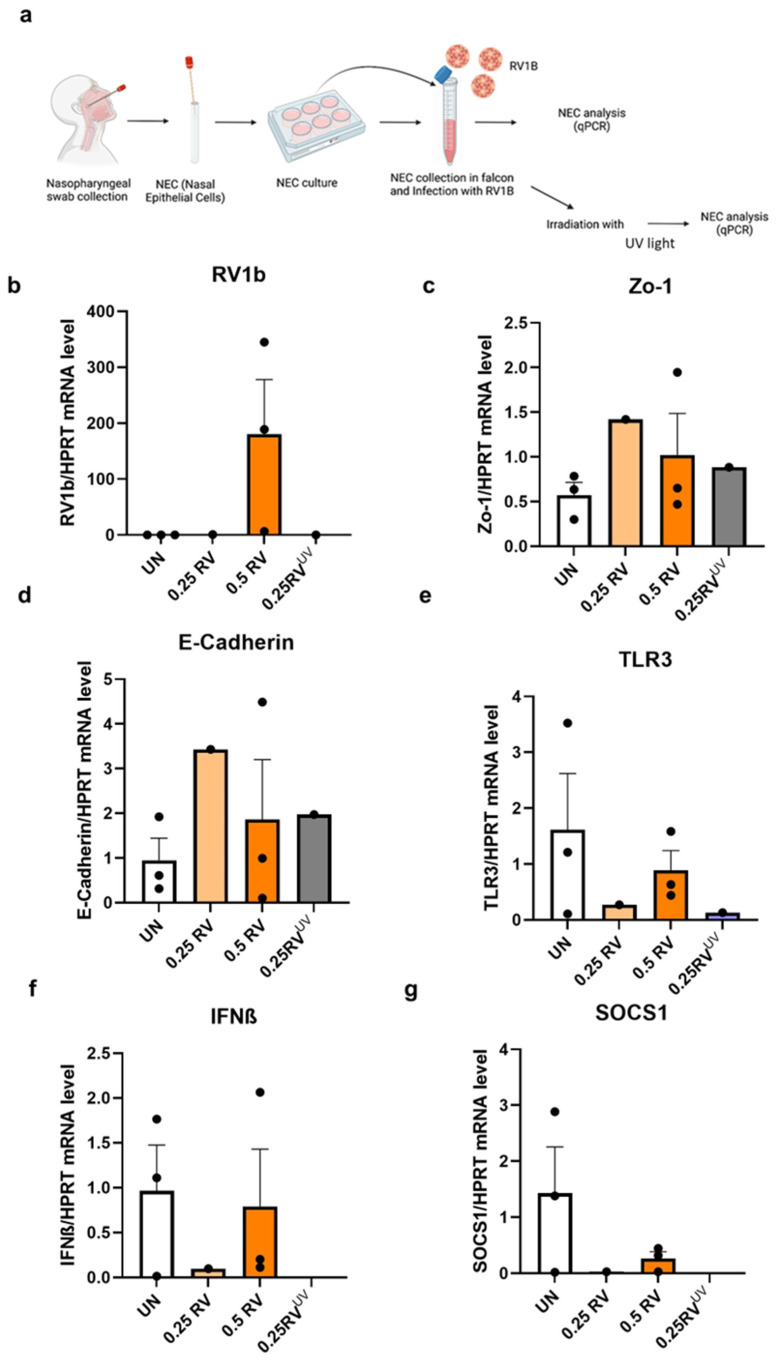
UV irradiation eliminated RV1B mRNA in nasal epithelial cells (NECs) but inhibited antiviral responses and cell integrity. (**a**) Experimental design of RV1B infection in NEC culture and UV irradiation. Created in BioRender.com. (**b**) Effects of RV infection in untreated and UV-irradiated RV-infected NECs at 48h. (**c**) Tight junctions in untreated and UV-irradiated RV-infected NECs measured within 48h. Each circular dot represents one independent sample per experiment and the number of dots per group corresponds to the sample size. Data represents three patients per group. (**d**) Expression of adherens junctions in untreated and UV-irradiated RV-infected NECs measured within 48h. Data represents three patients per group. (**e**) Viral sensing capability in untreated and IR-irradiated RV-infected NECs measured within 48 h. (**f**) Effects of antiviral response in untreated and UV-irradiated RV-infected NECs measured within 48h. (**g**) SOCS1 expression in untreated and UV-irradiated RV-infected NECs measured within 48 h. The mRNA levels of each condition were determined using real-time polymerase chain reaction. UV reduced the RV infection and inhibited the antiviral function after UV treatment.

We have analyzed the role of UV light on RV infection in NECs. Here, the NECs were either infected with RV1B and left untreated for 48 and 72 h or 0.25 mL RV irradiated with UV light for 30 min and then added with NECs, as indicated in Figure 5a. This treatment revealed that RV mRNA induces gradually in a dose-dependent manner after the infection in NECs, whereas it is reduced after UV treatment (Figure 5b). Next, the effect of RV and UV on the tight junction and adherens junctions was analyzed in NECs (Figure 5c,d). We did not observe any significant change after UV treatment, which demonstrated that UV did not influence tight and adherens junctions in NECs. Furthermore, the viral recognition capacity and interferon response after RV infection and UV treatment were analyzed via observing TLR3 expression and IFN response (Figure 5e,f). This data induced both TLR3 and IFN-β expression after RV infection and was reduced after UV treatment. Finally, to understand the inhibitory effect of interferon response, SOCS1 mRNA expression was analyzed after RV infection and UV treatment in NECs (Figure 5g). We found that SOCS1 was induced after RV infection but reduced after UV treatment.

### 3.6. UV Light Completely Eliminated RV1B mRNA in A549 Cells

**Figure 6:**
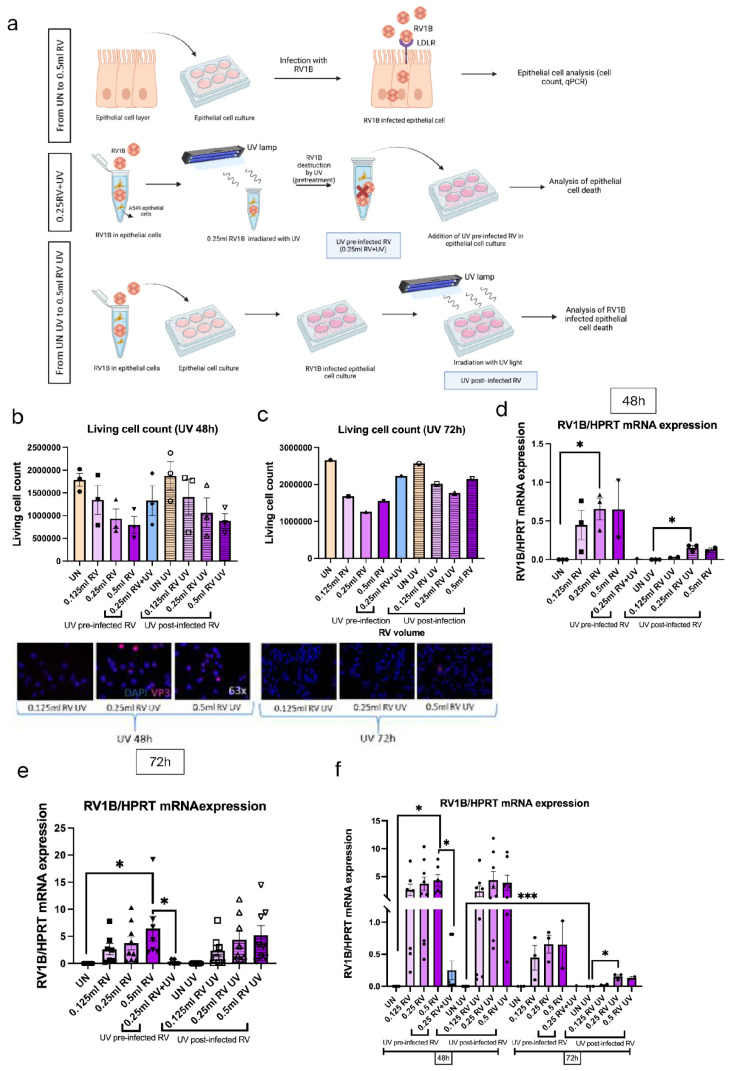
UV light eliminated RV RNA completely within 72 h after RV infection in lung tumor cell line A549. (**a**) Schematic illustration of RV1B infection in RV-infected, UV pre-irradiated and post-irradiated RV-infected airway epithelial cells. Created in BioRender.com. (**b**) Living A549 cell count within 48 h after UV light exposure with immunostaining results. Merged images shown (VP3 = pink; DAPI = blue). For (**b**), data is the representative of three different experiments. (**c**) Living cell count within 72 h under UV light with immunostaining results. Merged images shown (VP3 = pink; DAPI = blue). For (**c**), data is the representative of one experiment. (**d**) Effects on RV mRNA on untreated and UV-irradiated RV-infected A549 epithelial cells were measured within 48 h. Data represents six independent repeats per group, representative of three independent experiments. (**e**) Effects of untreated and UV irradiation on RV RNA in RV-infected A549 epithelial cells were measured within 72 h. Data represents three independent repeats per group, representative of one independent experiment. (**f**) Comparison between RV mRNA expression levels in untreated and under UV light within 48 h and 72 h. The mRNA levels of each condition were determined using real-time polymerase chain reaction. One-way ANOVA analysis with Tukey’s post hoc test was performed to test statistical significance. *p* values: * *p* < 0.05; *** *p* < 0.001. Each circular dot represents one independent sample per experiment and the number of dots per group corresponds to the sample size.

As IR treatment was not able to inhibit RV infection, UV light on RV infection in A549 cells was analyzed. A549 cells were either infected with increasing concentrations of RV1b and left untreated for 48 or 72 h or RV-infected and then treated with UV for 30 min, as indicated in Figure 6a. Moreover, as a positive control we infected A549 with RV-UV inactivated (UV pre-infected RV). Here we found that RV reduced by trend the living cells both in RV-infected untreated and UV-treated conditions at 48 h (Figure 6b). However, at 72 h we noticed that UV treatment counteracted RV-induced cell death (Figure 6c). Along with that, the immunostaining results also portrayed the same outcome where RV VP3 was visible in UV-treated conditions at 48 h but absent in UV-treated conditions at 72 h (Figure 6b,c).

RV1b mRNA expression was significantly induced and was directly proportional to the amount of RV infection in untreated cells at 48 h (Figure 6d). At 72 h the amount of RV1b mRNA decreased (Figure 6e,f). Considering UV 72 h treatment, we found that it eliminated the RV1b mRNA completely (Figure 6f). Taken together these data suggested that UV treatment eliminated RV in epithelial cells within 72 h, and this effect rescued epithelial cells from death.

### 3.7. RV Reduced the Adherens Junction in Epithelial Cells Within 48 h

**Figure 7:**
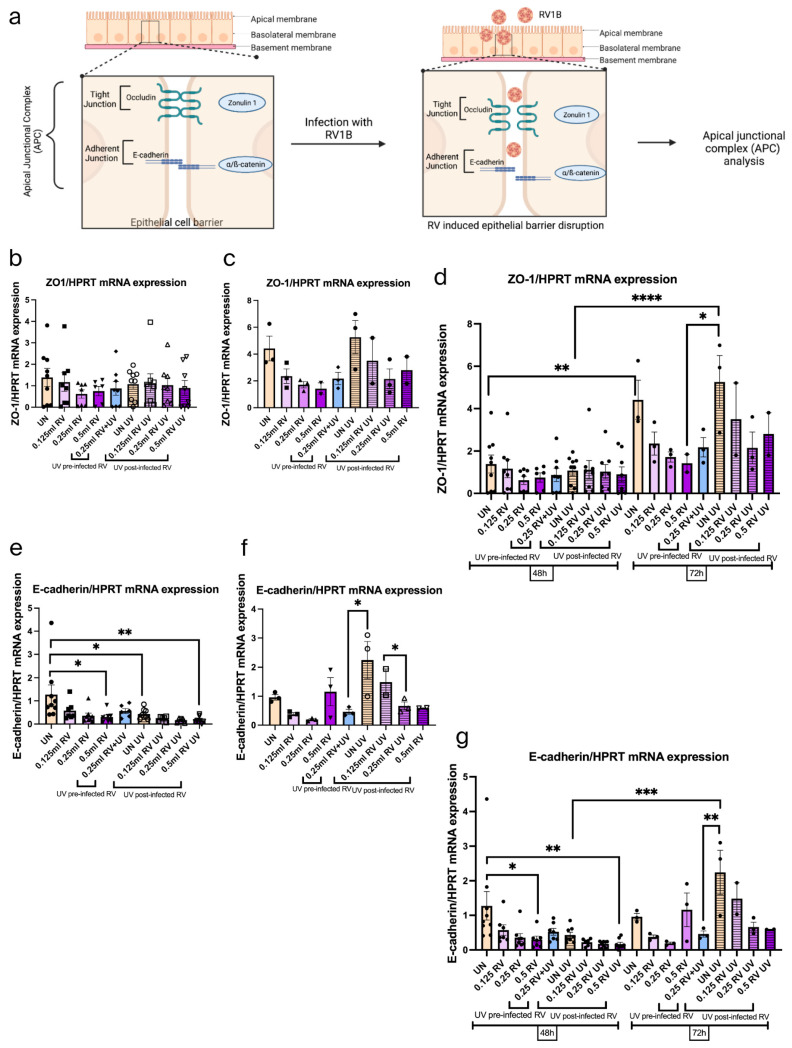
UV light reduced the adherens junction in epithelial tumor cells within 48 h after RV infection. (**a**) Schematic illustration of RV1B infection in the barrier junctional proteins of airway epithelial cells. Created in BioRender.com. (**b**) Effects on tight junctions in untreated and UV-irradiated A549 epithelial cells measured within 48 h. Data represents six independent repeats per group, representative of three independent experiments. (**c**) Effects on tight junctions in untreated and UV-irradiated RV infection in A549 epithelial cells measured within 72 h. Data represents three independent repeats per group, representative of one independent experiment. (**d**) Comparison between tight junctions in untreated and UV-irradiated RV infection within 48 h and 72 h. (**e**) Effects of adherens junctions in untreated and UV-irradiated RV infection in A549 epithelial cells measured within 48 h. Data represents six independent repeats per group, representative of three independent experiments. (**f**) Effects of adherens junctions in untreated and UV-irradiated RV-infected A549 epithelial cells measured within 72 h. Data represents three independent repeats per group, representative of one independent experiment. (**g**) Comparison between RV mRNA expression levels of adherens junction in untreated and under UV light within 48 h and 72 h. The mRNA levels of each condition were determined using real-time polymerase chain reaction. The adherens junction destruction occurred significantly after RV infection. One-way ANOVA analysis with Tukey’s post hoc test was performed to test statistical significance. *p* values: * *p* < 0.05; ** *p* < 0.01; ***p < 0.001; **** *p* < 0.0001. Each circular dot represents one independent sample per experiment and the number of dots per group corresponds to the sample size.

To explore the tight junction molecule, we first investigated ZO-1 mRNA expression levels under RV and UV effect (Figure 7a–d). Here we found a trend of reduced ZO-1 mRNA expression by increased RV concentrations, whereas an induction of ZO-1 was observed at UV 72 h treatment.

Moreover, to understand the function of adherens junctions we looked over the E-cadherin mRNA expression. This study showed significantly decreased E-cadherin mRNA expression by both RV and UV independently at 48 h (Figure 7e).

Furthermore, we found an additive inhibitory effect of RV and UV at 72 h post infection (Figure 7f,g). However, after UV 72 h treatment alone there was induced E-cadherin.

In summary, this data demonstrates that both RV and UV at 48 h have an inhibitory effect on adherens junctions.

### 3.8. Viral Sensing Capability by TLR3 Receptor and IFN-β Response Were Induced After RV1B Infection Within 72 h and Inhibited by UV Treatment

RV entry in the epithelial cells is initially sensed through pattern recognition receptors (PRRs), such as toll-like receptor 3 (TLR3). TLR3 responds to the presence of dsRNA, and signaling through TLR3 involves the activation of the nuclear factor kappa-light-chain-enhancer of activated B cells (NF-κB) whose pathway results in the release of inflammatory cytokines [43].

**Figure 8:**
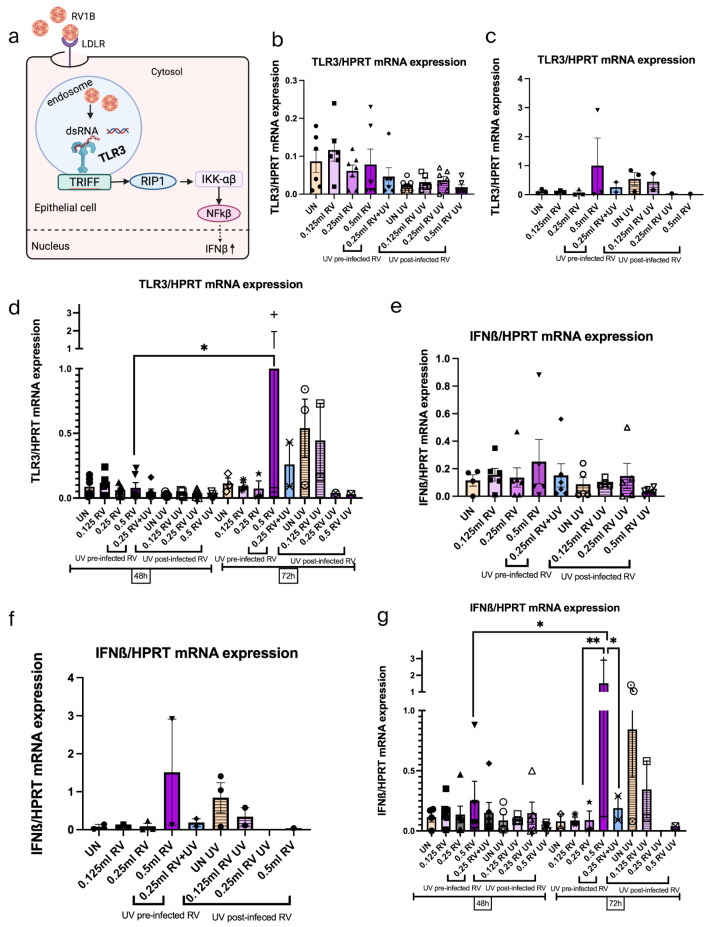
Viral sensing capability by TLR3 receptor and IFN-ß response were induced after RV1B infection within 72 h and inhibited by UV treatment. (**a**) Schematic illustration of RV1B infection in the viral recognition capability and antiviral response of airway epithelial cells. Created in BioRender.com. (**b**) Effects of viral sensing capability in untreated and UV-irradiated RV-infected A549 epithelial cells measured within 48 h. Data represents six independent repeats per group, representative of three independent experiments. (**c**) Effects of viral recognition in untreated and UV-irradiated RV-infected A549 epithelial cells measured within 72 h. Data represents three independent repeats per group, representative of one independent experiment. (**d**) Comparison between viral recognition capability in untreated and post UV light-treated conditions within 48 h and 72 h. (**e**) Effects of antiviral response in untreated and UV-irradiated RV-infected A549 epithelial cells measured within 48 h. Data represents six independent repeats per group, representative of three independent experiments. (**f**) Effects of antiviral response in untreated and UV-irradiated RV-infected A549 epithelial cells measured within 72 h. Data represents three independent repeats per group, representative of one independent experiment. (**g**) Comparison between antiviral response in untreated and UV light-treated RV infection within 48 h and 72 h. The antiviral defense mechanism was activated after RV1B infection within 72 h as both the viral recognition and interferon response were induced, whereas they were reduced after UV treatment. The mRNA levels of each condition were determined using real-time polymerase chain reaction. One-way ANOVA analysis with Tukey’s post hoc test was performed to test statistical significance. *p* values * *p* < 0.05; ** *p* < 0.01. Each circular dot represents one independent sample per experiment and the number of dots per group corresponds to the sample size.

In order to understand the recognition of RV infection and the induction of IFNs, at first, we investigated the role of the innate response of TLR3 to RV infection in A549 cells, the target cell for RV infection. Besides that, the effect of the UV light on TLR3 was also addressed. Secondly, we analyzed the interferon (IFN-β) expression to determine subsequent inflammatory response in the airways. Analysis showed that RV induced TLR3 at 72 h in a RV dose-dependent manner (Figure 8a–d). However, UV light inhibited this effect. A similar effect was observed on interferon response. Results from IFN-beta mRNA expression demonstrated increased IFN response after RV infection and decreased IFN response after UV 72 h treatment (Figure 8e–g). Collectively, this data demonstrates that RV induced antiviral pathways in the host cells, but UV inhibited antiviral effects of the host.

### 3.9. UV Light Induced the SOCS1 in UV-Irradiated Uninfected Epithelial Cells While Reducing SOCS1 in Case of RV-Infected Epithelial Cells Within 72 h

**Figure 9:**
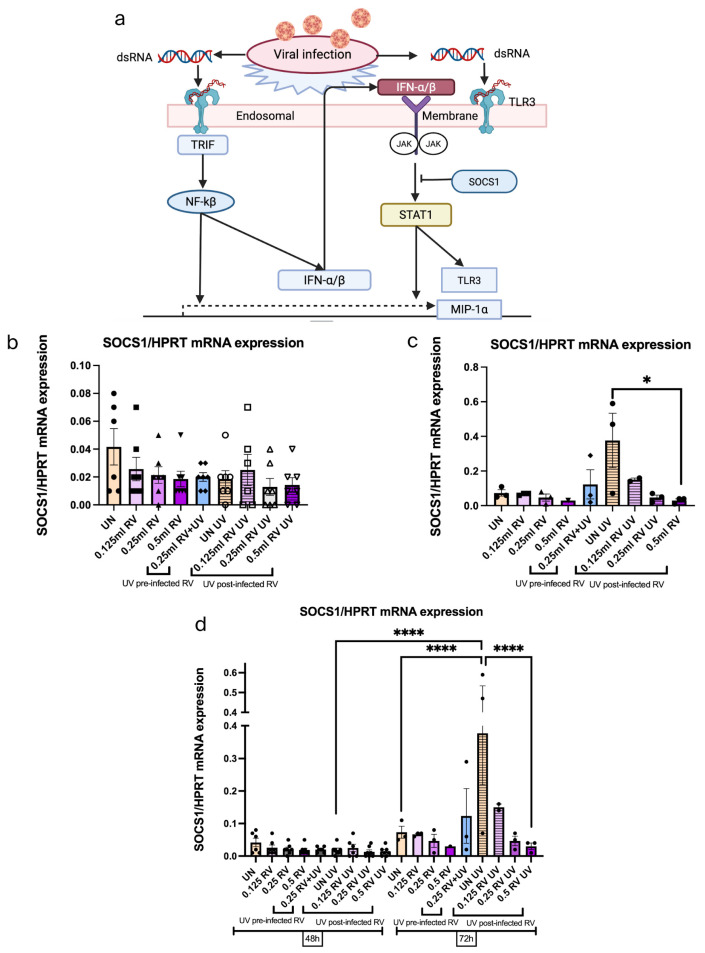
UV light induced SOCS1 mRNA in UV-irradiated uninfected tumor epithelial cells while reducing it in the case of RV-infected cells within 72 h. (**a**) Schematic illustration of the inhibitory effect of SOCS1 in the JAK/STAT pathway in airway epithelial cells after RV1B infection. Created in BioRender.com. (**b**) SOCS1 expression in untreated and UV-irradiated RV-infected A549 epithelial cells measured within 48 h. Data represents six independent repeats per group, representative of three independent experiments. (**c**) SOCS1 expression in untreated and UV-irradiated RV-infected A549 epithelial cells measured within 72 h. Data represents three independent repeats per group, representative of one independent experiment. (**d**) Comparison between SOCS1 mRNA expression levels in untreated and UV light post-treated conditions within 48 h and 72 h. The immune responses in RV-infected airway epithelial cells were reduced after UV 72 h treatment, which were higher in uninfected cells. The mRNA levels of each condition were determined using real-time polymerase chain reaction. One-way ANOVA analysis with Tukey’s post hoc test was performed to test statistical significance. *p* values * *p* < 0.05; **** *p* < 0.0001. Each circular dot represents one independent sample per experiment and the number of dots per group corresponds to the sample size.

In support of our previous findings, analysis of TLR3 expression and IFN response after RV infection demonstrated increased antiviral response (IFN-β) against RV by increasing innate immunity of host response. However, the suppressor of cytokine signaling (SOCS) 1 is a negative regulator of interferon production [44]. SOCS1 can block IFN function both by directly impairing transcription and by binding to the IFN receptor. To address this, we performed the analysis of SOCS1 mRNA expression under RV and UV treatment. We have found that the UV-irradiated uninfected epithelial cells showed the highest SOCS1 induction, whereas it was significantly reduced in RV-infected epithelial cells after UV 72 h treatment (Figure 9a–d). These findings demonstrate that UV 72 h treatment suppressed the natural immune response in uninfected epithelial cells as there are no RVs. Moreover, UV reduced the natural immune response of the RV-infected epithelial cells, as it was shown in our previous data that UV killed the RV within 72 h.

## 4. Discussion

**Figure 10:**
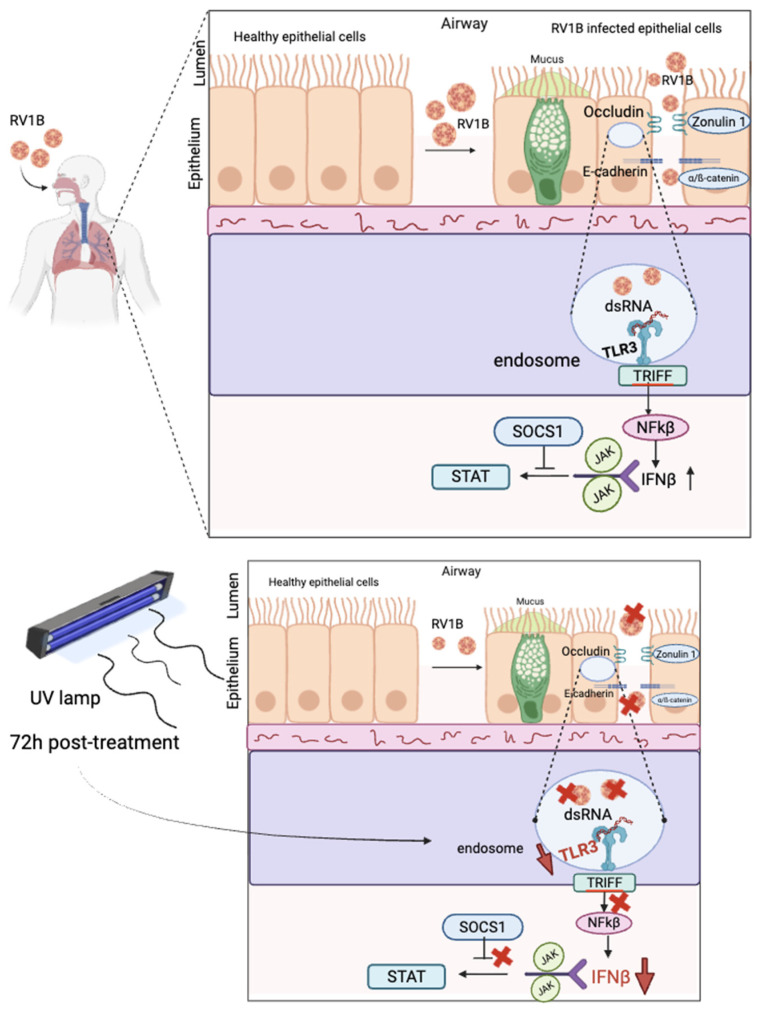
Model of RV1B effect in airway inflammation and the advantage of using UV and IR irradiation to destroy RV1B by reducing airway inflammation. (**Top**) Schematic shows the proposed destruction of airway epithelial tight junction and adherens junction destruction due to RV1B infection, which ultimately increases interferon-beta response. (**Bottom**) Panel shows the UV 72 h treatment advantage in destroying RV1B and altering RV1B affecting the apical junctional complex (tight junction and adherens junction) and interferon response. See text for further details. Created in BioRender.com.

In this study, we have analyzed an in vitro model about the ability of UV and IR lights to inactivate RV1B in epithelial cells and their resulting impact on epithelial barrier integrity and antiviral response (Figure 10).

Upon challenge with the triggering factor RV1B, our data demonstrate that RV1B causes the epithelial cell death. RV1B can directly act on the epithelium through impairment of barrier function [45]. Interestingly, we also identified downregulation of tight junction protein (ZO-1) and adherens junctions proteins (E-cadherin) after RV1B infection in epithelial cells. This alteration in junctional proteins of epithelial cell barriers increases the risk of asthma because of the barrier perturbation function by altering junctional proteins increases the paracellular permeability and allows easier viral invasion in airway epithelial cells [46].

Novel therapeutic targets for RV-associated diseases may be identified by understanding the basis of RV sensing and signaling leading to IFN and pro-inflammatory cytokines. Our study also showed the induction of TLR3 expression after RV infection within 72 h. Afterwards, interferon responses were investigated as TLR3 expression induces antiviral response by sending signals to the TRIF (TIR domain-containing adapter inducing IFN-β) and activating NF-κB pathway [47]. Notably, we also identified induced IFN-β expression after RV infection within 72 h.

Our time course analysis noted no significant change in RV1B inactivation in epithelial cells after UV 48 h treatment. Interestingly, UV 72 h treatment showed the highest RV1B inactivation in airway epithelial cells, which revealed UVC light is able to rescue airway epithelial cell death. Here, we observed the lowest RV mRNA expression even in the highest RV concentration, suggesting that UVC light causes dimerization in viral nucleic acid resulting in genetic damage of the virus. To inactivate the virus, chemical drugs that need to penetrate the virus membrane’s hard capsid may need several weeks to be effective against viruses, and may have side effects [37]. UVC avoids systemic exposure and does not depend on pathogen-specific drugs. This suggests that UVC light can be considered as a safe and less invasive physical antiviral approach than traditional antiviral therapy. The result depends on both the UVC dose and the virus concentration.

The observation of junctional proteins in epithelial cell barrier revealed that UV light reduces the adherens junction (AJ) in epithelial cells within 48 h, as a significant reduction of E-cadherin mRNA expression was observed after UV 48 h treatment. Though, no significant changes were observed in the case of tight junction and adherens junction (72 h). Our study on these findings demonstrates that UVC cannot improve epithelial barrier integrity after RV infection in airway epithelial cells as they are already destroyed by viral invasion. UVC light does not damage host biology, but cannot repair the cell damage after infection.

Our study also showed reduction in both TLR3 and IFN-β expressions after UV 72 h treatment. These findings revealed that viral sensing capability and antiviral response were reduced after UV 72 h treatment even in higher RV doses. This observation further ensures that UV inactivates the virus, and this may be the reason for decreased antiviral function in RV-infected airway epithelial cells. Our study on SOCS1 demonstrated increased SOCS1 expression in UV-treated uninfected epithelial cells but decreased SOCS1 expression after UV treatment in RV-infected epithelial cells. These findings revealed that UV inhibited antiviral effects of the host via SOCS1. Combined, these data suggest that ultraviolet radiation may have a role in inhibiting the expression and function of SOCS1 as a potential therapy against viruses and asthma exacerbators.

In our study, we observed that IR treatment could not inhibit the RV1B in A549 cells, but it could inhibit it in primary nasal epithelial cells. This might relate to the different origin of the A549, which is of alveolar type II-like epithelial cells, more representative of the distal lung alveoli. Besides this limitation, this finding indicates that the therapeutic use of the IR light can be used instead of UVC light to block RV1B in the upper airways’ epithelial cells, and it has the advantage to induce antiviral responses via TLR3 receptor and IFN-β response after 48 h IR treatment while reducing SOCS1 in RV-infected nasal epithelial cells within both 48 h and 72 h IR treatment. Non-ionizing light like IR cannot directly damage the virus’s DNA, but it does make atoms vibrate, potentially heating the virus [34]. This mild thermal effect of IR is a potential strategy to be considered for future studies.

Indeed, we have demonstrated that UV light has a superior antiviral activity as compared to IR light that can be a potential treatment in the context of severe viral load. However, this treatment decreased the epithelial barrier function as well as the antiviral immune responses of the host. Although it would be interesting to investigate the effect of UV and IR in inactivating other RV subtypes and extend the subject number in the NEC experiment, these results indicate a risk in repeated UV application, especially for the destruction of the epithelial barrier and the antimicrobial defense of the host. Moreover, it suggests that IR light provides a possible alternative to UV to limit the RV expansion at its entrance in the upper airways. In conclusion, we recognize that the major limitation of this work is the analysis of nasal epithelial cells derived from three subjects, and thus these analyses need to be extended to a larger cohort of control, allergic and asthmatic subjects. Moreover, additional work was performed in a cell line derived from the lower respiratory tract. Thus, separated and extended analysis of the effect of UV and IR lights should be done on upper and lower respiratory tract-derived epithelial cells.

However, this work points out at the cytopathic effects that the UV light has on the epithelial cells and the possible alternative to use IR light, although with the later having a lower impact on the rhinovirus elimination but preserving the integrity and the antiviral immune response.

Collectively, these findings support the role of UV light as an effective therapeutic approach for acutely eliminating RV, but resulting in barrier and antiviral damage, which can have a drawback effect for asthma.

## Figures and Tables

**Table 1:** Characteristics of the subjects whose NECs were analyzed in this study.

Patient ID	Sex	Allergic Asthma	Allergic Rhinitis	Pollen Allergy	Contact Allergy	Food Allergy	Animal Dander
1	Female	no	yes	no	yes	yes	no
2	Female	yes	no	yes	no	no	no
3	Female	no	no	no	no	no	yes

**Table 2:** List of the primer sequences used in this study.

Primer	Type	Sequence
HPRT	Fwd	CATTATGCTGAGGATTTGGAAAGG
	Rev	CTTGAGCACACAGAGGGCTACA
RLP30	Fwd	CTGGTGTCCATCAC TACAGTGG
	Rev	CCAGTCTGTTCTGGCATGCTTC
RV1B (also called RUVBL1)	Fwd	GAAGACAGAGGTGCTGATGGAG
	Rev	CTCTGTCTCACACGGAGTTAGC
ZO-1 (Encoded by TJP1)	Fwd	GTCCAGAATCTCGGAAAAGTGCC
	Rev	CTTTCAGCGCACCATACCAACC
E-cadherin	Fwd	GCCTCCTGAAAAGAGAGTGGAAG
	Rev	TGGCAGTGTCTCTCCAAATCCG
TLR3	Fwd	GCGCTAAAAAGTGAAGAACTGGAT
	Rev	GCTGGACATTGTTCAGAAAGAGG
SOCS1	Fwd	TTCGCCCTTAGCGTGAAGATGG
	Rev	TAGTGCTCCAGCAGCTCGAAGA
IFN-b	Fwd	CTTGGATTCCTACAAAGAAGCAGC
	Rev	TCCTCCTTCTGGAACTGCTGCA

## Data Availability

The original contributions presented in this study are included in the article/Supplementary Materials. Further inquiries can be directed to the corresponding author.

## Supplementary Materials

The following supporting information can be downloaded at https://www.mdpi.com/article/10.3390/v18030303/s1. Figure S1: Nasal Epithelial Cells (NEC) characterization before exposure. Around 70% of these nasal epithelial cells are EpCAM+ (upper panels), and of these 10% are also EGFR+ (lower panels). By contrast A549 are 100% EGFR+; Table S1: Consumables; Table S2: Laboratory equipment.

## Abbreviations

°C	Degree Celsius
AJ	Adherens junction
AJC	Apical junctional complex
BSA	Bovine serum albumin
bp	Base pairs
BC	Before Christ
CO2	Carbon dioxide
cm	Centimeter
CD	Cluster of differentiation
DNA	Deoxyribonucleic acid
dNTP	Deoxyribonucleotide triphosphate
DAPI	4′,6-diamidino-2-phenylindole
dsRNA	Double-stranded RNA
EDTA	Ethylenediamine tetraacetic acid
FCS	Fetal calf serum
g	Gram
h	Hour
HPRT	Hypoxanthine-guanine phosphoribosyl transferase
HDM	House dust mite
IgE	Immunoglobulin E
IR	Infrared radiation
IFN	Interferon
IFN-β	Interferon-beta
ml	Milliliter min Minute
mm	Millimeter
mRNA	messenger ribonucleic acid
nm	nanometer
NEC	Nasal epithelial cell
NIR	Near-infrared
pen	Penicillin
PFA	Paraformaldehyde
PBS	Dulbecco’s phosphate buffered saline
qPCR	quantitative polymerase chain reaction
RNA	Ribonucleic acid
RPL30	Ribosomal protein L30
RT	Room temperature
RV	Rhinovirus RV1B human rhinovirus 1B
RPMI	Roswell park memorial institute medium
rpm	Revolution per minute
sec	Second
SOCS1	Suppressor of cytokine signaling 1
TJ	Tight junction
TLR3	Toll-like receptor 3
Th1 cell	T-helper 1 cell
Th2 cell	T-helper 2 cell
UVC	Ultraviolet C
VP	Viral protein
ZO-1	Zonulin-1
μL	Microliter
