# Supplementary material for: UV Light Inhibited HRV1b Replication but Reduced Adherens Epithelial Junction and Antiviral Responses via SOCS1 in Human Respiratory Epithelial Cells
Source: Viruses. 2026 Feb 28;18(3):303. doi: 10.3390/v18030303 (PMC13030477; doi:10.3390/v18030303)
Supplement: Supplementary file 1 [file viruses-18-00303-s001.zip › viruses-4129333-supplementary.pdf]

Supplementary Table S1: Consumables

Product	Item number	Manufacturer
RPMI 1640 medium	Cat.: AC-LM-0060	Anprotec
DMEM medium	Cat.: AN-MS017M2	Anprotec
PBS	Cat.: AC-BS-0002	Anprotec
FBS Superior FCS	LOT: 0001647073	Sigma
Penicillin/Streptomycin (PenStrep)	Cat.: AC-AB-0024	Anprotec
L-Glutamine	Cat.: AC-AS-0001	Anprotec
Trypsin-EDTA	Cat.: AC-EZ-0001	Anprotec
Storage solution	LOT: 5210309327	Miltenyi Biotec
Quiazol Lysis Reagent	79306	Quiagen
Chloroform	67-66-3	VWR Chemicals
Isopropanol	CP41.3	Roth
Glycogen	HP51.1	Roth
5x Reaction Buffer	LOT: 01328324	Thermo Scientific

Product	Item number	Manufacturer
RPMI 1640 medium	Cat.: AC-LM-0060	Anprotec
DMEM medium	Cat.: AN-MS017M2	Anprotec
PBS	Cat.: AC-BS-0002	Anprotec
RiboLock RNase Inhibitor	LOT: 01314835	Thermo Scientific
dNTP Mix	LOT: 01051079	Thermo Scientific
Revert Aid Reverse Transcriptase	LOT: 01326638	Thermo Scientific
Random Hexamer Primer	LOT: 01294675	Thermo Scientific
SYBR green		
RV VP3 (mono/mouse)	MA5-18249	Invitrogen
AlexaFluor 555 (poly/mouse)	A-21425	Invitrogen
Fixation/Permeabilization Diluent	00-5223-56	Invitrogen
Permeabilization Buffer	00-8333-56	Invitrogen
FLuorSave Reagent (mounting Medium)	345789	Merck
Collagenase	C98991-500mg	Sigma-Aldrich

Supplementary Table S2: Laboratory equipment

**Product**	**Item number**	**Manufacturer**
Pipette tips (10, 200, 1000 µl)	Cat.: S1111-3000<br>Cat.: S1111-1006<br>Cat.: S1111-6001	Starlab Tipone ®
Pipetting aid<br>Accu-jet® -pro	REF: 26301	BRAND
Reaction vessel (0,5; 1,5; 2,0 ml)	Eppendorf	Order-no.: 0030 121.023<br>Order-no.: 0030 120.086<br>Order-no.: 0030 120.094
Well plates (6 well)	Cat.: 657 160	Greiner bio-one<br>CELLSTAR
Well plates (6 well)	Lot: 211225-074-B	Th. Geyer GmbH & Co.KG
Tubes (15, 50ml)	Cat.: 188271<br>Cat.: 227261	Greiner bio-one<br>CELLSTAR
Microscope slide (76×26 mm)	Order number: 021102	DIAGONAL Gmbh & Co.KG
Incubation shaker	00174/781	Edmund Buehler GmbH
Pipettes (2.5, 10, 20, 100, 200, 1000µl)	3123000012<br>3123000020<br>3123000098<br>3123000047<br>3123000055<br>3123000063	Eppendorf
Incubator	6161000	Edmund Buehler GmbH
Freezer	HFU300TV63	Thermo FIsher Scientific

Ultra low temperature freezer		New Brunswick
Vortexer	629334	Janke und Kunkel IKA Labortechnik
Nanodrop 2000C	Serial No. 1305	Peqlab
Master cycler	100199414	Peqlab
C1000™ thermal cycler	Serial no: 785BR70	BIO-RAD Laboratories
Microscope	Primo Vert	Zeis
Incubator	Type: BBD 6220<br>Serial-No.: 41205888	Thermo Scientific
Mini centrifuge	Serial no: HSA27955	Biozym
Liquid blocker		Science Services
Safety benches	00182/062	Scarlaf
Multiplate® PCR Plates™ 96 well, clear	Calalog: MLL9601	Bio-Rad Laboratories, Inc.
Microseal® 'B' seal	Catalog: MSB1001	Bio-Rad Laboratories, Inc.
Infrared light IR11	par38e	Petra electric
